# Final Adult Height in Girls Treated with GnRH Analogues for Precocious Puberty Around Age Eight †

**DOI:** 10.3390/children12060756

**Published:** 2025-06-11

**Authors:** Semra Bahar Akın, İlker Tolga Özgen

**Affiliations:** 1 Division of Pediatric Endocrinology, Department of Pediatrics, Health Sciences University Derince Training and Research Hospital, Derince 41100, Kocaeli, Turkey; 2 Division of Pediatric Endocrinology, Department of Pediatrics, Biruni University Hospital, Küçükçekmece 34290, Istanbul, Turkey; iozgen@biruni.edu.tr

**Keywords:** GnRHa treatment, final adult height, bone mineral density, body mass index, after 8 years old, precocious puberty

## Abstract

**Introductions:** Precocious puberty initiated at a very young age causes a severe loss in height potential and should be treated with gonadotropin-releasing hormone agonists (GnRHa). Controversial findings exist regarding the efficacy of GnRHa treatment in girls with central precocious puberty (CPP) onset around the age of 8. This research assessed the impact of GnRHa treatment on the final height (FAH) of 117 girls diagnosed with CPP within this age group. **Methods:** This retrospective study included 117 CPP girls diagnosed at around age 8 (7–9 years old). Girls who started treatment between the ages of 8 and 9 (*n* = 71) and 7 and 8 (*n* = 46) were divided into groups 1 and 2, respectively. Predicted height (PAH), target height (TH), and FAH were calculated from medical records. Girls’ PAH, TH, and FAH were also compared between groups. **Results:** At beginning of treatment, the girls’ average ages were 8.59 ± 0.27 in group 1 and 7.50 ± 0.47 in group 2. In groups 1 and 2, GnRHa therapy durations were 1.97 ± 0.54 and 2.91 ± 0.61, respectively. There were no significant differences in TH (160.53 ± 5.49 vs. 160.57 ± 4.94), PAH (158.72 ± 5.23 vs. 158.35 ± 5.57), and FAH (162.42 ± 5.32 vs. 162.14 ± 5.70) between groups. FAH improved 4 cm from PAH in both (*p* = 0.001). Multivariate linear regression analysis showed that baseline height SDS was the main FAH predictor (Beta: 0.572, *p* = 0.001). **Conclusions:** GnRHa may improve FAH even if the treatment is delayed after age 8. However, as this improvement is limited for this age group, the therapy option should be individualized and should not be considered for all children.

## 1. Introduction

Puberty commences with the activation of pulsatile hypothalamic gonadotropin-releasing hormone (GnRH), which subsequently triggers pituitary gonadotropin secretion and gonadal steroid synthesizing. Puberty can be affected by a variety of factors, including genetics, nutrition, environment, and socioeconomic status [1]. The exact mechanisms that affect the onset of puberty are unclear [2]. Precocious puberty is defined by the onset of breast development in girls before they reach 8 years of age [3].

One of the important goals of central precocious puberty (CPP) treatment is to conserve final adult height. Nonetheless, it should be acknowledged that certain patients may exhibit a non-progressive or slowly progressive variant of CPP, allowing them to attain normal adult height without intervention. Therefore, an observation period is usually appropriate prior to starting treatment. Moreover, some patients may be admitted to the hospital after the age of 8 with pubertal stage Tanner 3 or more. Thus, sometimes, the initiation of the treatment may be delayed. Several studies have shown that girls treated before the age of 6 achieve the greatest final height gain, while those with pubertal onset between 6 and 8 years may also benefit from treatment.. In contrast, it has been suggested that girls aged ≥8 years do not receive enough benefit from treatment in terms of height [4]. In this study, we investigated the effect of late-onset treatment on final adult height (FAH) in girls over 8 years old.

## 2. Methods

### 2.1. Study Design

This study was an observational retrospective cohort study performed on 117 girls with CPP who were diagnosed around the age of 8 (7–9 years old) from October 2011 to March 2015.

### 2.2. Study Groups and Objectives

The girls whose treatment was initiated between the ages 8–9 years (*n* = 71) and 7–8 years (*n* = 46) were included in the Groups 1 and 2, respectively. Exclusion criteria included the following: (1) chronic systemic diseases affecting growth (e.g.; hypothyroidism; celiac disease; chronic kidney disease); (2) prior or concomitant treatment with growth hormone; (3) premature birth (defined as <37 weeks of gestation); (4) peripheral precocious puberty preceding central activation (e.g.; non-classical congenital adrenal hyperplasia); and (5) incomplete clinical or auxological data.

The effect of the GnRH treatment on FAH was compared in groups who started the therapy after and before the age of 8. The predicted adult height (PAH) and FAH were also compared in groups. Finally, we compared FAH with TH in groups.

### 2.3. Diagnostic and Treatment Criteria

All patients had confirmed CPP based on the following criteria: breast development starting before the age of 8, luteinizing hormone (LH) peak over 5 IU/L following the GnRH test, and a difference greater than 1 year between bone age (BA) and chronological age (CA). Patients whose bone age was less than 1 year ahead were monitored for pubertal progression. Treatment was started for those who progressed rapidly.

GnRHa treatment for girls aged 8–9 years was initiated based on a synthesis of clinical and auxological indicators, including a rapid progression of secondary sexual characteristics (Tanner stage advancement within 6 months), bone age advancement ≥ 1.5 years above chronological age, and psychosocial stress [5].

### 2.4. Data Collection

Baseline CA, BA, growth velocity before treatment, mother and fathers’ heights (to calculate TH), weight standard deviation scores (SDS), height SDS and body mass index (BMI) SDS, basal LH, follicle-stimulating hormone (FSH), estradiol (E2), stimulated LH, FSH, and LH/FSH ratio were recorded. Moreover, CA, BA, weight SDS, height SDS, BMI SDS, LH, FSH, E2, the age of the menarche, and FAH were also recorded at the end of the follow-up.

A Harpenden stadiometer (Holtain Ltd., Crymych, Wales, UK) and a SECA scale (Seca GmbH & Co. KG., Hamburg, Germany) were used to measure standing height in centimeters, accurate to the nearest 0.1 cm, and body weight in kilograms, precise to the nearest 0.1 kg, respectively. Bone age was assessed via the Greulich and Pyle atlas [6]. The same person evaluated bone age during this study. PAH was determined using the Bayley–Pinneau method, which was applied to the height and bone age at the commencement of treatment [7]. BMI was calculated and represented as kg/m^2^. Cole’s least mean square approach was used to calculate BMI as a standard deviation score (BMI SDS) [8].

Baseline and stimulated LH and FSH levels, baseline serum E2 levels, thyroxine, TSH and 25-hydroxyvitamin (25(OH)-D) levels were assessed using immune–chemiluminescence (ARCHITECT i2000SR System, Abbott Laboratories, Abbott Park, IL, USA) [9]. The diagnostic limit for the estradiol assay was 10 pg/mL. Serum calcium was measured using the arsenazo III methodology, serum phosphor was measured using the phosphor phosphomolybdate method, and alkaline phosphatase (ALP) was measured using the para-nitrophenyl phosphate method (ARCHITECT c16000 System, Abbott Laboratories, Abbott Park, IL, USA).

For medical records, 52 of the 117 girls had data on bone health. Bone mineral density (BMD) measurements were available for patients who were followed in the bone health clinic or who underwent the technique of dual-energy x-ray absorptiometry (DXA) (GE Lunar Corp., Madison, WI, USA) for clinical indications. Serum calcium, phosphor, ALP, 25(OH)D, and BMD z-score at the sixth month after the end of the treatment were obtained from records.

BMD was assessed using DXA Lunar Corp. and. Hologic Discovery (Hologic, Inc., Waltham, MA, USA) at the lumbar spine (L1–L4). 

### 2.5. Treatment Protocol

All patients commenced therapy with leuprolide acetate 3.75 mg administered via intramuscular injection every 4 weeks. Height and weight measures, secondary sexual development, and gonadal hormone levels (LH, FSH, and E2) were assessed biannually. X-rays were performed annually. Cranial MRI was performed in children with precocious puberty only in the presence of neurological symptoms or rapid progress. If the growth rate was greater than 6 cm/year, secondary sexual development was rapid, and/or gonadal hormone levels were high, we changed the dose of leuprolide acetate to 7.50 mg intramuscular injection every 4 weeks [4]. GnRHa was terminated when BA or CA attained 12 years and 11 years, respectively.

Patients were monitored annually following the termination of medication, with measurements of height and weight measured. X-ray images were taken. The follow-up concluded upon reaching FAH. FAH is considered to be when BA is ≥15 years and/or the growth rate is below 2 cm annually (or within ≥2 years post-menarche for girls); TH is ascertained based on the height of each parent.

### 2.6. Statistical Analysis 

Results are presented as means ± SD. The variables between the groups before and after the age of 8 were compared using Student’s *t*-test. The variables PAH versus FAH and BMI SDS before versus at the end of the therapy were compared with paired *t*-test. *p* < 0.05 was considered statistically significant. Multivariate linear regression analyses (with the enter method) were performed to assess the major influencing factor of adult height. Baseline height SDS, target height, bone age advancement, BMİ, and treatment duration were included in the multivariate analysis. Data was analyzed applying the Statistical Package for the Social Sciences (SPSS), version 21.0 (IBM Corp., Armonk, NY, USA).

### 2.7. Ethics Approval

The local ethics committee approved this study (approval no: 2022/393; approval date: 27 September 2022) and was conducted in accordance with the Declaration of Helsinki. Informed consent was not required due to the nature of this retrospective chart review utilizing anonymized data, as permitted by the institutional review board.

## 3. Results

### 3.1. Final Height Outcomes and Predictors of Growth Response

The mean ages of the girls at the beginning of the treatment were 8.59 ± 0.27 in Group 1 and 7.50 ± 0.47 in Group 2. The means of TH, PAH, and FAH were not statistically different between the groups (Table 1). A comparison of the anthropometric and laboratory features of the groups is shown in Table 1. Furthermore, FAH improved by approximately 4 cm compared to the PAH in both groups (*p* < 0.001). All brain MRIs performed in selected patients were normal.

A multiple linear regression analysis was conducted to determine the independent predictors of FAH SDS. The independent variables included baseline height SDS, target height, baseline BMI SDS, age at treatment initiation, and treatment duration. The analysis demonstrated that baseline height SDS was the strongest predictor of FAH (β = 0.572, *p* < 0.001), indicating that initial growth status plays a key role in determining height outcomes. Target height also represented a statistically significant contribution (B = 0.049, *p* < 0.001). In contrast, baseline BMI SDS (*p* = 0.774), age at treatment initiation (*p* = 0.229), and treatment duration (*p* = 0.650) were not statistically significant predictors. These findings suggest that growth potential at the onset of treatment is more critical than treatment duration or age of initiation (Table 2).

### 3.2. Bone Assessment and Imaging

There were not any girls with osteoporosis after the treatment. However, 4/52 girls had a BMD z-score between −1 and −2. BMD measurements were available for patients who were followed in the bone health clinic or who underwent DXA for clinical indications; thus, this subgroup may not fully represent the entire cohort.

Serum calcium and phosphorus levels were measured in all patients and were found to be within normal reference ranges throughout the treatment period.

### 3.3. BMI Evolution

BMI SDS increased significantly during the first year of treatment (mean ± SD: 1.04 ± 0.67 vs. 0.97 ± 0.65, *p* = 0.049) and increased further by the end of treatment (1.10 ± 0.65, *p* = 0.002 vs. baseline). A notable decrease in BMI SDS was observed after treatment discontinuation, reaching 0.25 ± 1.12 at final adult height (*p* = 0.002 vs. baseline) (Table 3).

## 4. Discussion

The elimination of inhibitory signals on GnRH neurons and the prominence of the excitatory systems leads to central precocious puberty [10]. Children with precocious puberty are exposed to estrogen at an earlier age, which continues throughout their reproductive years [11]. It has been previously shown that earlier exposure to estradiol may lead to short stature, may increase some risks for cancer [12], and may have a negative effect on psychosocial health. A known risk factor for breast cancer is long-term estradiol exposure [13,14]. In addition, the psychological impact of PP on girls can be detrimental. There is a strong association between precociousness and negative psychological, behavioral, and social impacts, according to recent research [15,16,17]. The administration of the treatment is reassuring in terms of psychological results, given the worries about the negative psychological effects of girls’ precocious puberty. Moreover, one of the primary concerns associated with precocious puberty is its potential adverse effects on the FAH.

Some studies have shown a modest effect on height between the ages of 6 and 8 but almost no effect after the age of 8 [18,19]. The average height gain in our study was 4 cm, which is comparable to previously reported gains in selected patient populations undergoing GnRHa treatment [20], although other studies have reported variable outcomes depending on treatment timing and baseline characteristics [20,21]. Although previous findings have reported no improvement after the age of 8, our findings suggest that patients who started treatment after the age of 8 experienced a similar height increase in children at ages 6–8. Prior studies found that individuals with shorter treatment periods and younger onset had faster height growth [22]. In our study, we found that both individuals who started treatment late and those who started early treatment grew to approximately the same height.

A consensus exists regarding the advantages of treating children with CPP prior to age 6, but there are controversial results regarding height gain in children treated after the age of 6. In one study, Cassio et al. [19] found no significant difference in final height between untreated and treated girls aged 7.5–8.5 years. However, the study had limitations, including a small sample size and the absence of GnRH testing. Similarly, Bouvattier et al. [18] reported no significant FAH difference between treated and untreated girls aged 8.4–10 years. However, most participants were older than 9 years, potentially reducing the likelihood of meaningful height gain. Korkmaz et al. [23] also reported no significant height difference between treated and untreated girls aged 6–8 years. Their study, however, lacked age-stratified analysis and did not account for potential confounders through regression modeling. Franzini et al. [24], in a meta-analysis of controlled studies in girls aged 7–10 years, likewise found no significant FAH difference (mean difference: 0.50 cm; 95% CI: –0.72 to 1.73 cm), but the inclusion of a wide age range and undefined PAH estimation methods may have introduced heterogeneity.

In contrast, several recent studies have reported more favorable outcomes, particularly when treatment was initiated around age 8. Vargas Trujillo et al. [25] demonstrated an average PAH gain of approximately 5 cm in girls treated at or after age 7. Lee et al. [26] similarly found an FAH gain of about 3.9 cm in girls treated at a mean age of 8.2 years, with no significant difference in outcomes based on age at initiation. Micillo et al. [27] showed that girls treated before and after age 8 achieved comparable near-final heights, suggesting potential benefit even in later-onset cases. Lin et al. [28] observed that girls aged 8–10 years with early puberty gained an average of 4.13 cm after treatment and reached their target height. Kauli et al. [29] also reported improved FAH in patients diagnosed after age 8 compared to untreated controls. Saito et al. [30], who reported a modest yet significant height gain (~3 cm) in girls treated before age 9. Lee et al. [31] also supported these findings, reporting a 3.9 cm FAH gain with no significant difference in outcomes between those treated before or after age 8. Taken together, these studies indicate that GnRHa treatment may offer height-related benefits even when initiated after age 7, particularly in selected patients with advanced bone age, compromised PAH, or rapidly progressing puberty. Collectively, these studies support the notion that GnRHa therapy may still offer height-related benefits when initiated in late-onset CPP cases, provided that treatment is appropriately individualized.

Our study evaluated more specific and limited age groups, 7–8 and 8–9 years, compared to the studies in the literature, and it involved a larger number of patients. Based on the outcomes of our study, the FAH may be improved with GnRH analogs even if it was initiated at the ages of 7–8 or 8–9. Although the mean FAH gain of ~4 cm was statistically significant, its clinical relevance should be interpreted cautiously, particularly for girls with near-target PAH at baseline.

The lack of a treated control group limits the capacity of this study to determine the causal effect of GnRHa treatment on FAH. Although the longitudinal design provides beneficial observational data, it cannot exclude the possibility that some height results may have developed independently of the treatment. Furthermore, in real-world clinical practice, withholding treatment in rapidly progressing cases of CPP is often viewed as ethically problematic, making it difficult to establish randomized control groups.

Another issue about the GnRH treatment is its side effects. Studies that compare the increase in BMI before and after treatment are inconclusive, and some show that GnRHa therapy may increase the risk of obesity in CPP patients [32,33,34]. In our study, BMI SDS increased significantly during treatment, particularly during the first year and by the end of treatment. However, a significant reduction was observed after treatment discontinuation, suggesting a reversible effect of GnRHa therapy on BMI. Another long-term effect in patients using GnRHa is the controversial results regarding the reduction in BMD. Even though BMD decreased while receiving GnRHa medication, this could be reversed by taking calcium supplements [35]. Some publications report that BMD increases following the end of treatment [36]. Additionally, some research has shown that GnRHa treatment does not cause a reduction in BMD [37,38]. Our results showed no negative effects of GnRHa treatment on BMD, which is consistent with earlier research. In addition, normal calcium and phosphorus levels observed during treatment suggest that GnRHa therapy did not have a negative impact on mineral metabolism in our cohort. However, only 52 patients had BMD data available, and this subgroup may not reflect the entire cohort, as measurements were based on clinical referral or follow-up in the bone health clinic. This limits the generalizability of BMD-related findings and warrants cautious interpretation.

## 5. Limitations

One of the key limitations of this study is its retrospective design, which restricts our ability to draw causal inferences regarding the impact of GnRHa therapy. Since all participants received treatment, comparisons rely solely on within-group changes, which may be influenced by confounding variables such as spontaneous variations in growth potential and secular trends. Also, the mean period of GnRHa use was less than 2 years in many of the subjects. In addition, BMD was not evaluated for all patients.

## 6. Conclusions

In conclusion, according to our data, an improvement in PAH was observed even when GnRH agonist therapy was started after the age of 8. Moreover, a similar improvement in PAH was observed in both treatment groups. However, treatments should be individualized as these treatments provide minor benefits.

## Figures and Tables

**Table 1:** Description of variable comparisons between Group 1 and Group 2.

	Group 1 (*n* = 71)	Group 2 (*n* = 46)	*p*
Age (years)	8.59 ± 0.27	7.50 ± 0.47	<0.001
Bone age (years)	10.40 ± 0.85	9.39 ± 1.03	<0.001
BA–CA difference (years)	1.75 ± 0.71	1.88 ± 0.96	0.35
Height (cm)	136.26 ± 5.66	130.08 ± 6.83	<0.001
Height SDS	0.81 ± 0.78	0.76 ± 0.78	0.711
Weight (kg)	36.09 ± 7.01	30.56 ± 4.84	<0.001
Weight SDS	1.12 ± 0.71	1.03 ± 0.58	0.470
BMI (kg/m^2^) (at baseline)	19.32 ± 2.82	18.49 ± 3.04	0.136
BMI SDS (at baseline)	1.02 ± 0.67	0.90 ± 0.59	0.314
Basal LH (mIU/mL)	1.36 ± 2.25	0.40 ± 0.66	0.007
Basal FSH (mIU/mL)	4.51 ± 2.37	2.88 ± 1.45	0.001
Basal E2 (pg/mL)	29.01 ± 21.53	19.25 ± 10.78	0.005
Stimulated LH peak (mIU/mL)	15.74 ± 10.72	12.49 ± 10.2	0.156
Stimulated FSH peak (mIU/mL)	14.53 ± 4.35	17.57 ± 6.78	0.013
Stimulated LH/FSH ratio	1.05 ± 0.61	0.87 ± 0.71	0.210
Number of patients receiving 7.5 mg dose GnRHa	5 (7%)	2 (4.3%)	_
Duration of the treatment (years)	1.97 ± 0.54	2.91 ± 0.61	<0.001
Mean age at treatment stop	10.63 ± 0.47	10.57 ± 0.50	0.45
Difference between treatment end and menarche (years)	1.08 ± 0.79	1.31 ± 0.91	0.138
Age of menarche (years)	11.72 ± 0.65	11.91 ± 0.70	0.176
Target height (cm)	161.26 ± 4.39	160.53 ± 5.49	0.447
Predicted adult height (cm)	158.49 ± 5.30	158.19 ± 5.69	0.774
Final adult height (cm)	162.63 ± 5.20	162.12 ± 5.42	0.627

BMI: Body mass index; SDS: standard deviation score; LH: luteinizing hormone; FSH: follicle stimulating hormone; E2: estradiol. BA: bone age; CA: chronological age; GnRHa: gonadotropin-releasing hormone analog.

**Table 2:** Results of multiple linear regression analysis for predictors of final adult height SDS.

Variable	Beta	*p*-Value
Baseline Height SDS	0.572	<0.001
Target Height	0.276	<0.001
BMI SDS	−0.019	0.774
Bone Age at Treatment Initiation	−0.109	0.229
Treatment Duration	−0.038	0.650

BMI: Body mass index; SDS: standard deviation score.

**Table 3:** Comparison of BMI SDS during treatment and at final height.

	BMI SDS (Mean ± SD)	*p*-Value vs. Baseline
Before Treatment	0.97 ± 0.65	–
First Year of Treatment Φ	1.04 ± 0.67	0.049
End of Treatment ϑ	1.10 ± 0.65	0.002
At Final Height Ω:	0.25 ± 1.12	0.002

Φ: Comparison of BMI SDS before treatment and at the first year of treatment (*p* < 0.049); ϑ: comparison of BMİ SDS before treatment and at the end of treatment (*p* < 0.002); Ω: comparison of BMİ SDS before treatment and at final height (*p* < 0.002). BMI: body mass index; SDS: standard deviation score.

## Data Availability

The data that support the findings of this study can be obtained from the corresponding author upon reasonable request.
